# Suicide

**Published:** 1868-12

**Authors:** 


### SUICIDE.

"*Self-slaying*," *falling by one's own hand,* is the literal meaning of a term, which, by common consent, is regarded as a crime against ourselves, against society, and against the Great Maker of us all. And yet in these latter days, it has found its advocates, like Congressional ruffianisms, polygamy, public plunderings, and the like. In a former number of the *New York Observer*, a communication from a clergyman appears, seconded by an editorial remark, "*Self-Killing, not Self-Murder.*" And the sentiment, so shocking in itself, has passed unrebuked. Has it come to this, that one of the most conservative religious papers in the whole country, can stand out, unreproved, as the palliator of one of the gravest crimes which a human being can perpetrate, inasmuch, as it is one that is not repented of? We can scarcely believe, that any man in his right mind, can kill himself; nor, properly speaking, can a man commit any sin at all, if he were in his right mind. A drunkard fires his neighbor's barn, or strikes his wife to the earth, and in his rage beats her to death. In his right mind, he would not have done so. But common consent sends him to the penitentiary or the gallows, without a question, because he put himself out of his right mind by his own act, by the indulgence of his own appetites; and just as guilty, do we beg leave to say, is the man, who, greatly gifted, abandons himself to study, to abundant eating, and a total neglect of those means of health, which our Maker, in his mercy, has placed within the reach of all. We are accountable for the right use of the reason which has been placed within us for our guide. Going mad after study, is not less a crime, than going mad after liquor, or after any other appetite; both are equally the unrestrained indulgence of a passion; one is the passion for brandy—the other is the passion for study. Are religious editors asleep, that they should allow to go unrebuked, an apology for self-destruction? We pause for answer.

